# Phase I/Ib study of olaparib and carboplatin in heavily pretreated recurrent high-grade serous ovarian cancer at low genetic risk

**DOI:** 10.18632/oncotarget.26869

**Published:** 2019-04-23

**Authors:** Erika J. Lampert, John L. Hays, Elise C. Kohn, Christina M. Annunziata, Lori Minasian, Minshu Yu, Nicolas Gordon, Tristan M. Sissung, Victoria L. Chiou, William D. Figg, Nicole Houston, Jung-Min Lee

**Affiliations:** ^1^ Women’s Malignancies Branch, Center for Cancer Research, National Cancer Institute, Bethesda, MD, USA; ^2^ Ohio State University, Columbus, OH, USA; ^3^ Genitourinary Malignancies Branch, Center for Cancer Research, National Cancer Institute, Bethesda, MD, USA

**Keywords:** high-grade serous ovarian cancer, BRCA wild type, olaparib, carboplatin

## Abstract

**Purpose:** To investigate maximum tolerated dose (MTD), activity and predictive biomarkers of olaparib with carboplatin in *BRCA* wild-type (*BRCA*wt) high grade serous ovarian carcinoma (HGSOC) patients.

**Methods:** A 3+3 dose escalation study examined olaparib capsules (400 mg twice daily [BID], days 1–7) with carboplatin (AUC3-5 on day 1) every 21 days for 8 cycles, followed by olaparib 400 mg BID maintenance. Blood and tumor biopsy samples were collected pre- and on-treatment in the expansion cohort for PAR levels and proteomic endpoints.

**Results:** 30 patients (median 7 prior regimens [2–12], 63% (19/30) platinum-resistant) were enrolled. Dose-limiting toxicity was thrombocytopenia/neutropenia, and infection with carboplatin AUC5 (2/6 patients). MTD was olaparib 400 mg BID + carboplatin AUC4. Grade 3/4 adverse events (>10%) included neutropenia (23%), thrombocytopenia (20%), and anemia (13%). Five of 25 (20%) evaluable patients had partial response (PR; median 4.5 months [3.3–9.5]). Clinical benefit rate (PR + stable disease ≥4 months) was 64% (16/25). A greater decrease in tissue PAR levels was seen in the clinical benefit group versus no benefit (median normalized linear change −1.84 [−3.39– −0.28] vs 0.51 [−0.27– 1.29], *p* = 0.001) and a DNA repair score by proteomics did not correlate with response.

**Conclusions:** The olaparib and carboplatin combination is tolerable and has clinical benefit in subsets of heavily pretreated *BRCA*wt HGSOC, independent of platinum sensitivity.

## INTRODUCTION

High grade serous ovarian cancer (HGSOC) is the most lethal gynecologic malignancy in the United States (U.S.), with an overall 5-year survival rate under 40% [1]. More than 80% of HGSOC present at an advanced stage, and recurrence is nearly universal leading to incurable disease [2]. Approximately 40% of HGSOC harbors defects in genes involved in homologous recombination (HR) repair (HRR) for double-stranded DNA breaks, such as *BRCA1* and *BRCA2* [3, 4]. While approximately 15% of HGSOC patients have a germline mutation in *BRCA1* or *BRCA2* (g*BRCA*m) [4, 5], HRR deficiency may be caused by other molecular alterations such as *RAD51C, RAD51D, BRIP1*, and *CHEK2* [6–8]. Deficient HRR leads to activation of alternate DNA repair pathways including base excision repair (BER) and non-homologous end-joining (NHEJ), which require poly (ADP-ribose) polymerase (PARP) activity [9]. Increased PARP-1 activity is demonstrated in several tumor types including HGSOC [10, 11]. In preclinical studies, PARP inhibitors (PARPi) showed enhanced cytotoxicity in both HGSOC with *BRCA* mutations and HRR-deficient HGSOC with wild-type *BRCA* (*BRCA*wt), suggesting a broad applicability of PARPi in clinic [12–14].

There are three PARPi - olaparib, rucaparib and niraparib - now U.S. Food and Drug Administration (FDA)-approved for ovarian cancer treatment or maintenance. Olaparib is the first approved PARPi for use as monotherapy in g*BRCA*m patients with recurrent HGSOC who had >3 prior treatment regimens [15]. It is also FDA-approved as a maintenance therapy in platinum-sensitive recurrent HGSOC patients regardless of their *BRCA* status, as well as for frontline maintenance in *BRCA*m patients [16–19]. Rucaparib, another PARPi, is approved for third-line treatment in patients with a germline or somatic *BRCA* mutation as well as for maintenance therapy [20, 21]. Like olaparib and rucaparib, niraparib is approved as a maintenance therapy in platinum-sensitive recurrent HGSOC patients who achieved response following chemotherapy [22]. So far, clinical benefits of PARPi in HGSOC appear strongest in *BRCA*m women in the platinum-sensitive recurrent treatment setting (response rates around 40–60%) [20, 23, 24]. Less activity is observed in heavily pretreated and non-*BRCA* mutant patients (response rates around 10–30% for platinum-sensitive and <10% for platinum-resistant) [20, 23], establishing the need to test combination strategies for this population.

Cisplatin, and now preferentially carboplatin, are the backbone of ovarian cancer treatment. Platinum agents form DNA-platinum adducts that damage DNA leading to cell death [25]. This is counteracted by the DNA repair mechanisms of BER and nucleotide excision repair [25–27]. Increased levels of poly(ADP-ribose) (PAR) polymers have been shown after cisplatin treatment in O-342 rat ovarian tumor cell lines [28] and PARP upregulation after cisplatin exposure was also demonstrated in normal renal tubular and human colon carcinoma cells [29, 30]. Concomitant use of PARPi with a platinum agent has been tested in several types of cancer, demonstrating increased cytotoxicity [31–35]. PARP inhibition potentiated platinum cytotoxicity in the O-342/DDP and CH1cisR platinum-resistant ovarian cancer cell lines [31, 32], as well as in the *BRCA*wt and *BRCA*2-restored OV90 and PEO4 ovarian cancer cell lines, respectively [33]. The PARPi CEP-6800 and olaparib also enhanced platinum-induced cytotoxicity in HRR*-*proficient non-small cell lung and colorectal carcinomas, respectively [34, 35]. These data support clinical testing of the PARPi and carboplatin combination in non-*BRCA* mutant ovarian cancer patients to assess for an additive or synergistic benefit of the doublet.

We previously reported the safety data and recommended phase 2 doses (RP2Ds) of olaparib in combination with carboplatin for patients with g*BRCA*m recurrent HGSOC or breast cancer and *BRCA*wt recurrent triple negative breast cancer (TNBC), finding different maximum tolerated doses (MTDs) for each population [36, 37]. We expanded these findings to women with HGSOC who do not have g*BRCA*m and now report safety, RP2D and activity. We also evaluated potential predictive and pharmacodynamic biomarkers for the PARPi and carboplatin combination.

## RESULTS

### Patients

**Figure 1:**
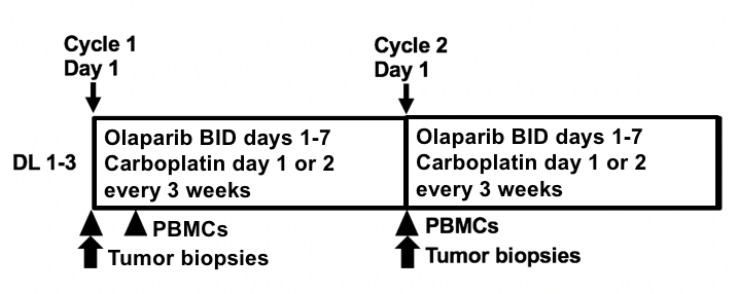
Study Schema. Abbreviations: DL: dose level; bid: twice daily; PBMCs: peripheral blood mononuclear cells.

**Figure 2:**
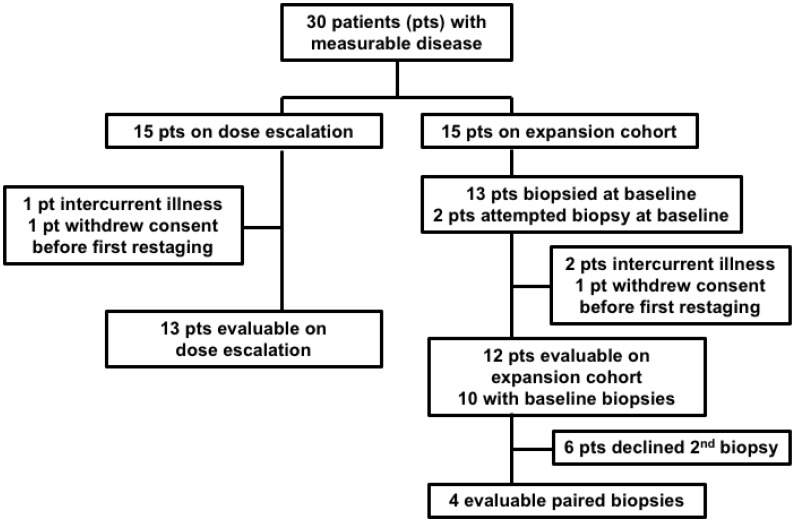
Consort Diagram. Of the 13 expansion cohort patients successfully biopsied at baseline, 10 had evaluable disease, 2 were non-evaluable due to intercurrent illness, and 1 withdrew consent. 6 of the 10 evaluable patients with a baseline biopsy declined a second biopsy, leaving 4 evaluable paired biopsies. Both of the non-evaluable patients with a baseline biopsy had a post-treatment biopsy, resulting in 2 non-evaluable paired biopsies for a total of 6 paired biopsies.

The study schema is depicted in Figure 1 and the Consort diagram is in Figure 2 (*N* = 30). All but 6 patients had negative deleterious g*BRCA*m commercial testing. Six patients enrolled between 2009–2013 had no BRCA testing prior to enrollment, and were eligible per protocol based on the negative family history of breast and ovarian cancer and a BRCAPro score of ≤20% [38]. Approximately two-thirds had platinum-resistant disease (63% [19/30]). Most patients (90% [27/30]) were heavily treated, having received more than three prior regimens. Other patient characteristics are shown in Table 1.

**Table 1:** Patient characteristics (*N* = 30)

Age in years, median (range)	65 (49–71)
ECOG Performance Status, *N* (%)	
0	5 (17%)
1	24 (80%)
2	1 (3%)
Median number of prior regiments (range)	7 (2–12)
Median prior chemotherapeutic agents (range)	6 (2–10)
Median prior biologic agents (range)	1 (0–3)
Prior bevacizumab treatment, *N* (%)^*^	21 (70%)
Prior vaccine treatment, *N* (%)	3 (10%)
Median months since last platinum (range)	16.5 (7–154)
Platinum sensitivity^+^, *N* (%)	
Platinum resistant recurrent disease	19 (63%)
Platinum sensitive recurrent disease	11 (37%)
Race/Ethnicity, N (%)	
White	27 (90%)
Black	2 (7%)
Asian	1 (3%)
Hispanic	0 (0%)

^*^Of patients with prior bevacizumab treatment, 62% (13/21) had platinum-resistant disease.

^+^Platinum sensitive: recurs 6 or more months after cessation of platinum-based chemotherapy; platinum resistant: progression within 6 months of platinum-based therapy

## Dose optimization

Patients received olaparib 400 mg capsules twice daily on days 1–7 and carboplatin AUC 3–5 on day 1 of each 21-day cycle (Table 2 and Figure 1). Olaparib 400 mg twice a day maintenance therapy was continued after a maximum of 8 carboplatin-containing cycles. No dose-limiting toxicities (DLT) were observed at dose level 2 (DL2) with carboplatin AUC4 during the 2-cycle evaluation period. Increasing to DL3 with carboplatin AUC5 resulted in 2 of 6 patients having DLT (grade 3 thrombocytopenia and neutropenia after one cycle [*n* = 1] and two concurrent grade 3 infections with an absolute neutrophil count (ANC) within normal range requiring IV antibiotics [*n* = 1]). One patient in DL3 required carboplatin dose reduction to AUC4 at cycle 4 for persistent neutropenia and treatment delays despite pegfilgrastim supplementation. Another DL3 patient was put on olaparib maintenance therapy after carboplatin discontinuation at cycle 6 due to neutropenic fever. No patients required olaparib dose reduction or (peg)filgrastim supplementation during maintenance therapy. The recommended phase 2 dose is olaparib capsules 400 mg twice daily days 1–7 with carboplatin AUC4 day 1 in 21-day cycles.

**Table 2:** Dose levels (*N* = 30)

Schedule and Dose				
Dose Level [DL]	Olaparib capsule, BID	Carboplatin IV, q3weeks	DLT	Best response (duration of response)
DL 1 (*N* = 3)	400 mg, days 1–7	AUC3, day 1 or 2	0	1 PR (7.5 mo) 2 SD (3 mo, 3 mo)
DL2 (*N* = 6)^*^	400 mg, days 1–7	AUC4, day 1 or 2	0	2 PR (3.3 mo, 4.5 mo) 2 SD (5.0 mo, 7.8 mo) 1 PD (2.4 mo) 1 NE (intercurrent illness)
DL 3 (*N* = 6)	400 mg, days 1–7	AUC5, day 1 or 2	2	1 PR (9.5 mo) 4 SD (8.5mo, 9.3mo, 10.8mo, 11.8mo) 1 NE (withdrew consent)
Expansion cohort (*N* = 15)	400 mg, days 1–7	AUC4, day 1 or 2		1 PR (4 mo) 7 SD (3.0mo, 3.5mo, 4.0 mo, 4.2 mo, 4.8 mo, 5.5mo, 10.6 mo) 4 PD (1.5 mo, 1.8 mo, 1.8 mo, 2.4 mo) 3 NE (1 withdrew consent; 2 intercurrent illness)

Abbreviations: bid: twice daily; mo: months; PR: partial response; SD: stable disease; PD: progressive disease; NE: non-evaluable.

^*^Six rather than three patients were enrolled in DL2 despite the absence of DLTs because the third level was added later.

## Adverse Events

Treatment-related adverse events (AEs) are summarized in Table 3. Hematologic toxicity was the most common AE (Tables 3, 4). Neutropenia occurred in 20 out of 30 patients (67%), with grade 3 or 4 neutropenia observed in 7 of 30 (23%) including one episode of febrile neutropenia. Ten of 30 (33%) patients received (peg)filgrastim to prevent treatment delay during the combination treatment. Grade 3 or 4 thrombocytopenia was observed in 6 of 30 patients (20%), and 1 patient (3%) required platelet transfusion after cycle 4 for grade 4 thrombocytopenia without bleeding. Grade 3 or 4 anemia occurred in four patients (13%) during cycles 2, 3, 4 and 5, respectively for each patient. Common (>10%) non-hematologic events included gastrointestinal side effects (nausea 50%, vomiting 27%, dyspepsia 30%), fatigue (33%) and headache (23%), and were predominantly grade 1 or 2, self-limited, and manageable with standard treatments.

**Table 3:** Drug-related adverse events by maximum grade per patient (*N* = 30)

Adverse Event	Grade 1	Grade 2	Grade 3	Grade 4	Grade 3/4
Hematology^*^					
Lymphocytopenia	4 (13%)	6 (20%)	11 (37%)	1 (3%)	40%
White Blood Count	4 (13%)	16 (53%)	6 (20%)	1 (3%)	23%
Neutropenia	2 (7%)	11 (37%)	3 (10%)	4 (13%)	23%
Thrombocytopenia	13 (43%)	4 (13%)	4 (13%)	2 (7%)	20%
Anemia	6 (20%)	16 (53%)	3 (10%)	1 (3%)	13%
**Gastrointestinal disorders**					
Mucositis	3 (10%)	1 (3%)	0	0	0%
Nausea	13 (43%)	2 (7%)	0	0	0%
Vomiting	5 (17%)	3 (10%)	1 (3%)	0	3%
Dyspepsia	8 (27%)	1 (3%)	0	0	0%
Gastroesophageal reflux disease	2 (7%)	0	0	0	0%
Constipation	6 (20%)	2 (7%)	0	0	0%
Diarrhea	4 (13%)	0	0	0	0%
**Chemistry**					
Hyponatremia	11 (37%)	0	0	0	0%
Hypokalemia	5 (17%)	0	1 (3%)	0	3%
Hypomagnesemia	11 (37%)	2 (7%)	1 (3%)	0	3%
Increased AST	5 (17%)	2 (7%)	0	0	0%
Increased ALT	8 (27%)	0	0	0	0%
**Other**
Fatigue	7 (23%)	3 (10%)	0	0	0%
Skin rash	3 (10%)	0	0	0	0%
Headache	7 (23%)	0	0	0	0%
Neuropathy^**^	4 (13%)	0	0	0	0%

^*^14 patients (47%) required blood transfusion. 4 patients (13%) received darbepoetin to avoid dose reduction or treatment delay.

^**^1 of 4 patients had baseline grade 1 neuropathy unchanged with treatment. 3 patients experienced treatment-related recurrence of grade 1 neuropathy.

**Table 4:** Drug-related hematologic adverse events by dose level (*N* = 30)

Adverse event	Grade 1			Grade 2			Grade 3			Grade 4		
	DL1	DL2	DL3	DL1	DL2	DL3	DL1	DL2	DL3	DL1	DL2	DL3
Lymphopenia	1(33%)	3(14%)	0	2 (67%)	3 (14%)	1 (17%)	0	8(38%)	3 (50%)	0	1 (5%)	0
Leukopenia	1(33%)	3(14%)	0	2 (67%)	11 (52%)	3 (50%)	0	3(14%)	3 (50%)	0	1 (5%)	0
Neutropenia	1(33%)	1(5%)	0	1 (33%)	8 (38%)	2 (33%)	0	2(10%)	1 (17%)	0	1 (5%)	3 (50%)
Thrombocytopenia	3(100%)	8(38%)	2(33%)	0	4 (19%)	0	0	1(5%)	3 (50%)	0	1 (5%)	1 (17%)
Anemia	2(67%)	3(14%)	1(17%)	0	12 (57%)	4 (67%)	0	2(10%)	1 (17%)	0	1 (5%)	0

Abbreviations: DL = dose level; Dose level 1: olaparib 400 mg bid days 1–7 with carboplatin AUC3; dose level 2 and expansion cohort: olaparib 400 mg bid days 1–7 with carboplatin AUC4; dose level 3: olaparib 400 mg bid days 1–7 with carboplatin AUC5

## Clinical activity

**Figure 3:**
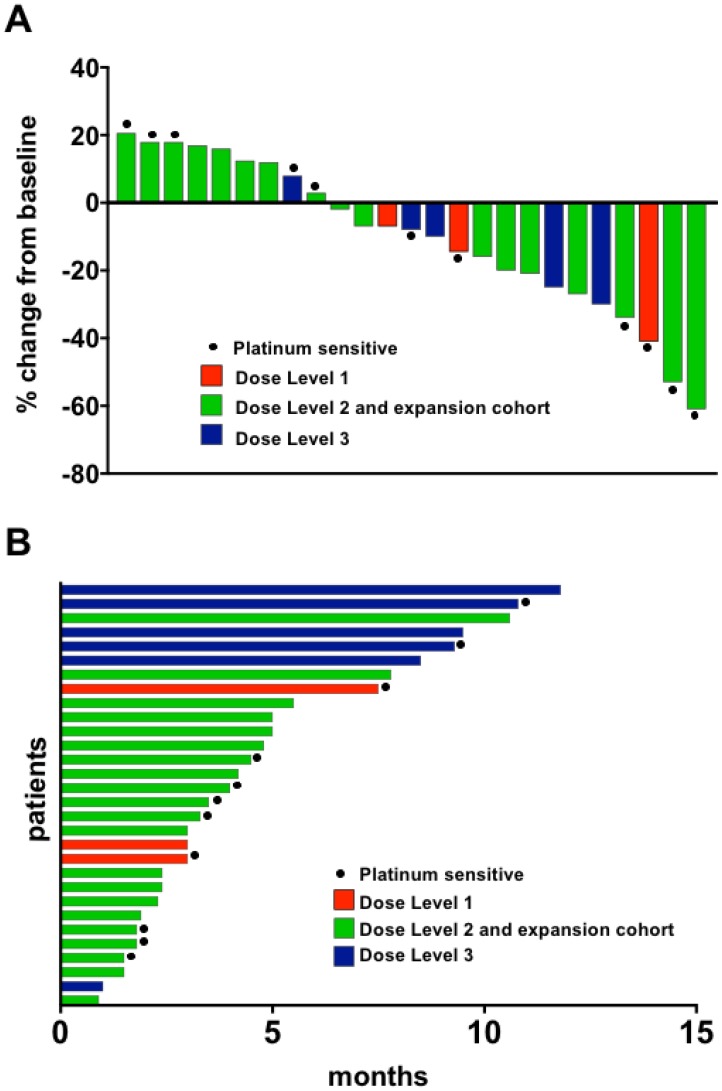
Waterfall plot (**A**) and duration on the study (**B**). (A) Twenty-five patients with baseline and subsequent imaging reassessment are shown. Best RECIST response is graphed for each patient. Five patients did not have reassessment scans for comparison (withdrew consent [*n* = 2], and off-treatment due to intercurrent illness [*n* = 3]). (B) All 30 patients are shown in a swimmer plot. Color code defines dose level of treatment. Dose level 1: olaparib 400 mg bid days 1–7 with carboplatin AUC3; dose level 2 and expansion cohort: olaparib 400 mg bid days 1–7 with carboplatin AUC4; dose level 3: olaparib 400 mg bid days 1–7 with carboplatin AUC5.

Twenty-five patients had disease evaluable for RECIST response determination (Figure 3A and 3B). Five patients attained a PR (median 4.5 months, range 3.3–9.5) yielding an overall response rate (ORR) of 20% (Table 5). 15 patients had stable disease (SD; median 5 months, range 3–11.8) for a clinical benefit rate (CBR, defined as PR+SD≥4 months) of 64% (16/25). Median progression-free survival (PFS) in the 25 evaluable patients was 4.2 months [1.5–11.8 months]. ORR was 7% (1/14) in platinum-resistant and 36% (4/11) in platinum-sensitive disease (Table 6). One patient with platinum-resistant disease who achieved PR (9.5 months) had 9 prior therapies. Nine patients with platinum-resistant disease also had SD ≥ 4 months, for a CBR of 71% (10/14). In platinum-sensitive disease, 4 patients achieved PR and 2 SD ≥ 4 months yielding a CBR of 55% (6/11; Table 6).

**Table 5:** RECIST response (*N* = 25 with evaluable disease)

Best Response	#(%)	Median Duration, mo (range)
**CR**	0	NA
**PR**	5 (20%)	4.5 (3.3–9.5)
**SD ≥ 4 mo**	11 (44%)	7.8 (4.0–11.8)
**Total SD**	15 (60%)	5 (3.0–11.8)
**PD**	5 (20%)	1.8 (1.5–2.4)
**Clinical Benefit Rate (CR+ PR + SD)**	20/25 (80%; for all SD) 16/25 (64%; for SD ≥ 4 mo)	
**Overall Response Rate (CR+PR)**	5/25 (20%)	

Abbreviations: pts: patients; mo: months; CR: complete response; PR: partial response; SD: stable disease; PD: progressive disease; NA: not applicable.

**Table 6:** RECIST response by platinum-sensitivity

Best Response	Platinum Sensitive (*N* = 11 evaluable disease)		Platinum Resistant (*N* = 14 evaluable disease)	
	**#(%)**	**Median Duration, mo (range)**	**#(%)**	**Median Duration, mo (range)**
**CR**	0	NA	0	NA
**PR**	4 (36%)	4.3 (3.3–7.5)	1 (7%)	9.5
**SD ≥ 4 mo**	2 (18%)	10.0 (9.3–10.8)	9 (64%)	5.5 (4.0–11.8)
**Total SD**	4 (36%)	6.4 (3.0–10.8)	11 (79%)	5.0 (3.0–11.8)
**PD**	3 (27%)	1.8 (1.5–1.8)	2 (14%)	2.4
**CBR (CR+ PR + SD)**	8/11 (73%; for all SD) 6/11 (55%; for SD ≥ 4 mo)		12/14 (86%; for all SD) 10/14 (71%; for SD ≥ 4 mo)	
**ORR (CR + PR)**	4/11 (36%)		1/14 (7%)	

Abbreviations: pts: patients; mo: months; CR: complete response; PR: partial response; SD: stable disease; PD: progressive disease; CBR: clinical benefit rate; ORR: overall response rate; NA: not applicable.

## Correlative studies

### PAR levels

**Figure 4:**
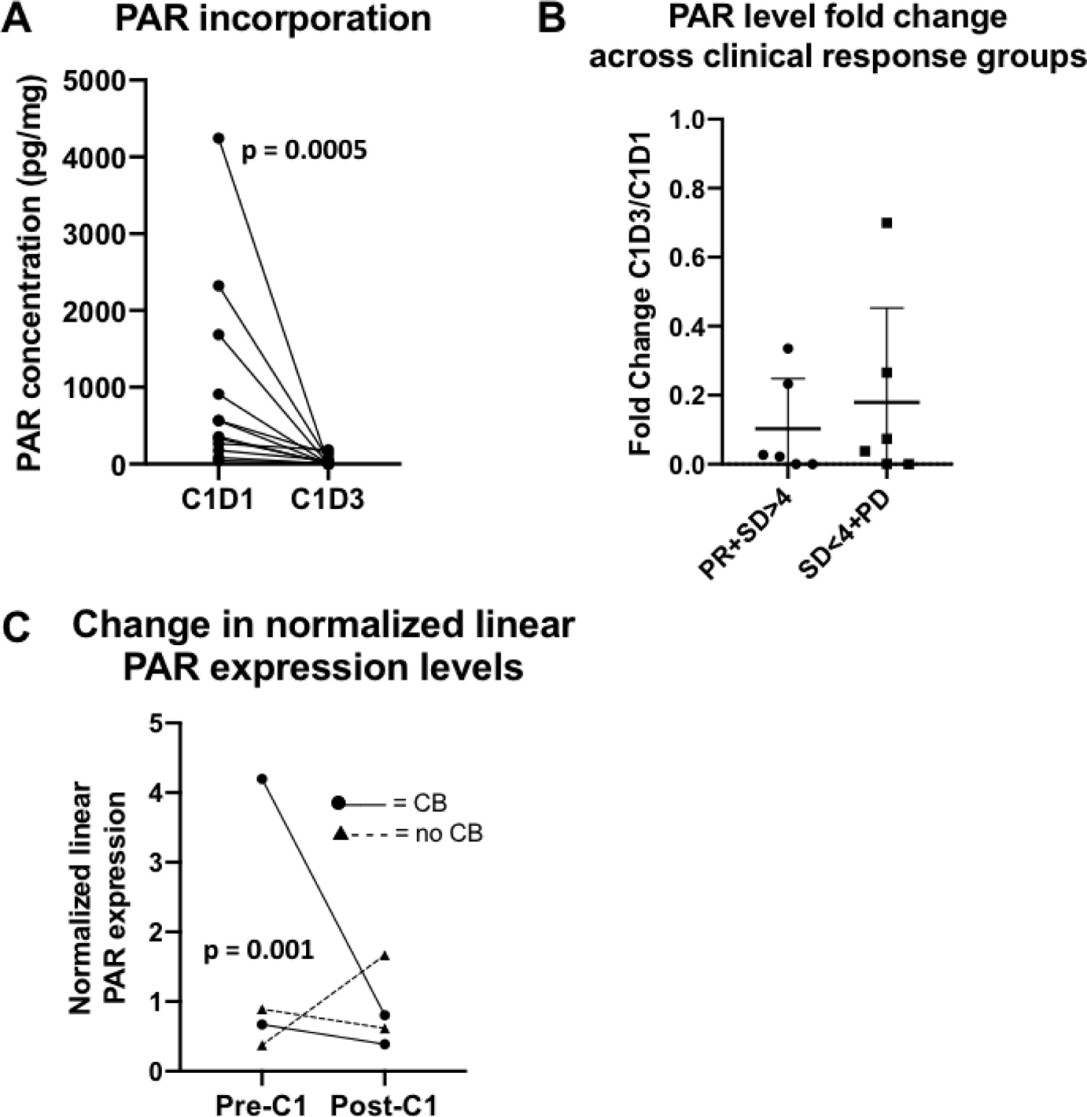
PAR translational studies. (**A**) PAR incorporation in peripheral blood. There was an expected decrease in PAR levels after treatment with olaparib in 12 evaluable paired samples. (**B**) Fold change in PAR concentration in peripheral blood by clinical response. No significant differences between fold change of PAR concentrations (C1D3/C1D1) and clinical response, defined as PR+SD>4 months versus SD<4 months+PD were observed. Abbreviations: C1D1 = cycle 1, day 1; C1D3 = cycle 1, day 3; PR = partial response; SD = stable disease; PD = progressive disease. (**C**) Change in normalized linear PAR expression levels between pre- and post-cycle 1 biopsy. A greater decrease of PAR levels was seen in the clinical benefit group (PR+SD≥4 months) versus no benefit group (SD<4 months+PD), *p* = 0.001. Abbreviations: C1 = cycle 1; CB = clinical benefit.

We first assessed the target effect induced by PARP inhibition as measured by PAR incorporation in peripheral blood mononuclear cells (PBMCs) and PAR expression levels in tissue biopsies. There was a significant decrease in PBMC PAR levels after treatment with olaparib in 12 evaluable paired samples (pre-treatment median 459.76 pg/mL [45.3–4, 243.5] vs post-treatment median 10.19 pg/mL [0–185.6], *p* = 0.0005; Figure 4A), demonstrating that olaparib reached pharmacologically effective concentrations. No correlation was seen between fold change from baseline to cycle 1 day 3 PAR incorporation and RECIST response (Figure 4B).

Of the 4 evaluable patients with paired biopsies, two had SD (4.2 and 10.6 months, respectively) and two had PD. A greater decrease in the normalized linear PAR expression levels was seen in the clinical benefit group (median change in normalized linear expression -1.84 [−3.39–−0.28]) compared to no benefit (0.51 [−0.27– 1.29]) (Figure 4C).

### PARP1/XRCC1 polymorphisms

No *PARP1* or *XRCC1* polymorphisms were associated with RECIST response or clinical benefit.

### Reverse phase protein microarray (RPPA) and a DNA repair score

Protein expression or protein post-translational modifications were assessed by RPPA [39]. Our modified version of the DNA repair score reported by Cardnell et al [40], incorporating baseline expression levels of 11 of the 17 originally used repair proteins that predicted PARPi sensitivity in small cell lung cancer, did not correlate with PFS (Supplementary Table 1; Supplementary Figure 1) [40].

Separately, the relationship between clinical response and pretreatment expression of all 274 proteins or post-translationally modified proteins on the RPPA array at the time was examined. Seven of 10 evaluable patients in the expansion cohort for whom baseline biopsies were obtained achieved clinical benefit and 3 patients had progressive disease. A false discovery rate (FDR) of 10% was used to control for multiple comparisons. Pretreatment biopsies were analyzed for linear correlation to PFS for each patient, without findings of significance in this small cohort.

### Tissue apoptotic index

Terminal deoxynucleotidyl transferase dUTP nick end labeling (TUNEL) assay was done on the available paired biopsies to evaluate DNA damage after treatment. There was no significant difference in mean apoptotic index before (47.9% [24.3–62.2]) and after (50.9% [28.09–75.61]) cycle 1 treatment with combination therapy (Supplementary Figure 2).

## DISCUSSION

This phase I/Ib study identified the schedule of olaparib, 400 mg capsules twice daily for days 1–7, administered with carboplatin AUC4 every 3 weeks as tolerable and providing modest activity in heavily pretreated *BRCA*wt HGSOC patients, including a majority who had platinum-resistant disease. A 400 mg twice daily olaparib capsule dose corresponds to a 250 to 300 mg twice daily olaparib tablet dose [41]. Consistent with our previously reported cohort of *BRCA*wt triple negative breast cancer patients, the intermittent olaparib schedule resulted in dose-limiting myelotoxicity, requiring a lower carboplatin dose (AUC4) than recommended for g*BRCA*m patients (AUC5) [36, 37]. This difference may be partly because more heavily pretreated patients were enrolled in the current cohort (median 7 prior regimens [2–12]) compared with the g*BRCA*m cohort (median 5 prior regimens [2–11]) [36].

The addition of PARPi to cytotoxic chemotherapy has previously been hindered by overlapping marrow toxicity, limiting the dose or treatment duration of PARPi and/or chemotherapy [42–45]. We had to reduce the standard carboplatin dose by over 50% in our phase I study of g*BRCA*m breast and ovarian cancer patients due to early hematologic toxicity with continuous daily olaparib, which led to successful use of the intermittent olaparib schedule [36]. We found manageable myelotoxicity with carboplatin and intermittently-dosed olaparib in the current cohort. Common (>10%) grade 3 and 4 toxicities were all hematological and managed with appropriate supplementation. Importantly, no grade 5 events were observed. Common non-hematologic adverse events, including gastrointestinal side effects were expected given the known and overlapping toxicities of each drug, and were predominantly grade 1 or 2 and either self-limited or addressed with standard care. These results align with a phase I trial of olaparib with cisplatin in advanced solid tumors that also required intermittent and lower dose olaparib (50–100 mg capsules days 1–5 or 1–10) with cisplatin every 21 days due to marrow toxicity [42]. Similarly, after a phase I trial of olaparib with carboplatin and paclitaxel reported that continuous olaparib intensified hematologic toxicities [45], a phase II trial used intermittent olaparib dosing (200 mg capsules days 1–10) with paclitaxel (175 mg/m^2^ every 3 weeks) and carboplatin (AUC4 every 3 weeks) in recurrent ovarian cancer patients for better tolerability and no clear loss of activity due to the reduced carboplatin dose [44]. Our results support the safety and tolerability of intermittent olaparib with carboplatin for combination therapy in *BRCA*wt recurrent HGSOC patients.

Platinum resistance is associated with a poor prognosis for women with ovarian cancer and almost all patients with recurrent disease ultimately develop resistance to platinum-based therapy [46]. Historically, the use of single agent cytotoxic chemotherapy or targeted therapy has resulted in RRs of around 5–15% and a median PFS of 3–4 months in the heavily pretreated, platinum-resistant patient population [47–52]. Our cohort of *BRCA*wt, heavily pretreated, largely platinum-resistant patients, thus represents a particularly difficult-to-treat population. In our study, 20% of patients achieved PR and two thirds had clinical benefit, although patients were heavily pretreated with a median of 7 prior therapies. We noted comparable clinical benefit in the patients with platinum-resistant (CBR 71%) and platinum-sensitive (CBR 55%) disease. It is possible that this clinical benefit is in part related to the required at least 6-month interval from last platinum exposure to initiation of protocol therapy.

It has been demonstrated preclinically that platinum-resistant clones present in the original tumor persist after platinum-sensitive clones are eradicated by chemotherapy [53, 54]. An *in vitro* study of the cisplatin-resistant A2780-CR human ovarian cancer cell line showed that following initial cisplatin exposure, cells became significantly more sensitive to cisplatin reintroduction after a 4-week drug-free interval, suggesting that platinum-resistant clones can be sensitized in a time-dependent manner [55]. Clinical studies also demonstrated that extending the interval from last platinum exposure in platinum-resistant or refractory ovarian cancer patients increases the likelihood of response to platinum retreatment [56, 57]. These data suggest re-challenging with platinum-based therapy may yield clinical activity in some platinum-resistant patients, thus the PARPi and carboplatin combination may be a therapeutic opportunity independent of platinum sensitivity. Future preclinical and clinical studies are warranted to explore this possibility.

Identifying and validating effective predictive biomarkers remains a challenge in the *BRCA*wt population. Among four evaluable paired biopsies, a greater decrease in PAR expression levels was observed in patients who achieved clinical benefit from treatment compared to those who did not, and this should be interpreted with caution due to small numbers. A measure of DNA repair deficiency has been widely investigated as a potential biomarker for the response to PARPi, and proteomic analysis has been employed to study functional DNA repair deficiencies [58, 59]. We applied a modified version of the “DNA repair protein score” developed by Cardnell *et al.* [40], consisting of expression levels of 17 DNA repair proteins shown to predict response to the PARPi BMN673 (talazoparib) in an *in vitro* small cell lung cancer model, and found no correlation to PFS. While the RPPA analysis used in the Cardnell study included 193 total and/or phosphorylated proteins, only 11 of the original 17 DNA repair proteins overlapped in the 274-antibody panel used here. Thus, we had to calculate a modified DNA repair score by taking the average expression levels of the overlapping 11 DNA repair proteins which may partly explain the lack of correlation. Also, genomic HR deficiency (HRD) was assessed in the ARIEL2 phase 2 trial of the PARPi rucaparib using next-generation sequencing combining *BRCA* mutation status with a measure of loss of heterozygosity (LOH) [20]. Both *BRCA*m and *BRCA*wt patients with high LOH had improved PFS and objective responses to PARPi treatment compared to BRCA*wt/*LOH-low patients (hazard ratio 0.27, *p* < 0.0001 and 0.62, *p* = 0.011 for *BRCA*m and LOH high subgroups, respectively, compared to LOH low) [20]. Similarly, the phase 3 NOVA study of niraparib maintenance therapy assessed HRD as a biomarker using the myChoice test from Myriad Genetics and found HRD-positive *BRCA*wt patients derived greater benefit from therapy versus placebo compared to the overall non-g*BRCA*m cohort (hazard ratio 0.38, *p* < 0.001 versus 0.45, *p* < 0.001, respectively) [22]. Further studies are warranted to identify predictive biomarkers of PARPi-combination treatment response in the BRCA-proficient population.

Our study has several limitations. First, our primary objective was to determine a safe dose and schedule in this patient population. Thus, we had insufficient power, even with an expansion cohort for the purpose of pharmacodynamic correlative exploration, to make conclusions of clinical benefit. We controlled for multiple comparisons to reduce the incidence of type 1 errors, however, due the exploratory nature of the translational endpoints all findings should be examined and validated prospectively before definitive conclusions can be made. We also did not prospectively plan to sequence genes involved in HRR and were unable to do so because of insufficient clinical samples remaining, and lack of proper consent for germline testing. Instead, HRR function was explored by assessing DNA repair protein expression, activation, and an experimental composite score.

There are now a number of PARPi combination studies in phase I to III clinical trials in ovarian cancer, though primarily focusing on the platinum-sensitive and *BRCA*m population. Our findings present an opportunity to further investigate a PARPi and carboplatin combination in heavily-pretreated *BRCA*wt patients. The observed modest activity of this combination, regardless of platinum sensitivity, supports the potential for platinum re-sensitization and highlights the need for continued biomarker analysis.

## MATERIALS AND METHODS

### Patients

Accrual occurred between 2009 and 2014. Eligible patients had recurrent or refractory HGSOC with known negative g*BRCA*m testing or a negative family history and a BRCAPro score of ≤20% calculated on the basis of detailed family history [38], an ECOG score of 0–2 and normal organ and bone marrow function. There was no limit on number of prior therapies, although patients had to be at least six months from last platinum exposure. Patients previously treated with a PARPi were excluded from the study although patients with platinum-resistant ovarian cancer were eligible. All patients provided written informed consent. This study was approved by the Institutional Review Board of the Center for Cancer Research, National Cancer Institute. ClinicalTrials.gov identifier: NCT01445418.

### Drug administration and determination of MTD

This open label 3+3 dose escalation study examined the combination of olaparib 400 mg capsules every 12 hours on days 1–7 with carboplatin AUC3, 4, or 5 on day 1 (DLs 1–3), in 21-day cycles (Figure 1). Carboplatin infusion was allowed on day 2 for those with schedule conflicts. No more than 8 cycles of combination therapy were given, followed by continuous daily maintenance olaparib monotherapy with 400 mg capsules every 12 hours. Safety and adverse events were graded every cycle (CTCAEv3.0). DLTs were defined as grade 4 hematologic and grade 3 or 4 nonhematologic adverse events related to study medications during the first two cycles of therapy. Grade 3 neutropenia for ≥7 days or with fever, and grade 3 thrombocytopenia lasting ≥7 days or requiring transfusion were also dose-limiting. Exceptions included grade 3 diarrhea, nausea, or vomiting, which had to be unresponsive to optimal medical care, and asymptomatic grade 3 reductions in electrolytes readily reversed with medical management. Complete blood counts and serum chemistries were monitored weekly during the DLT period. MTD was defined as the highest dose level at which ≤33% of patients experienced a DLT.

Granisetron (days 1–7) and dexamethasone (days 1–4) were given prophylactically for nausea during each combination therapy cycle and discontinued during olaparib maintenance. (Peg)filgrastim was not permitted during the first 2 cycles of the dose escalation phase but was indicated for use in subsequent cycles if the day 1 ANC was 1500–1800/mL or if the day 1 ANC was less than 1500/mL, necessitating treatment delay. Once initiated, (peg)filgrastim was continued during all combination treatment cycles, but was not used during olaparib maintenance therapy.

Clinical response was assessed by imaging using RECISTv1.0 criteria every 2 cycles. Study treatment was discontinued for progression of disease, intercurrent illness, adverse events not recovering to grade 1 within 3 weeks, and patient preference.

### Correlative studies

PBMCs were collected at baseline and cycle 1 day 3 for PAR incorporation (Figure 1). PBMCs were separated within 4 hours of collection, then stored in aliquots at –80° C until use. PBMC DNA PAR incorporation was measured with a commercial PAR immunoassay (Trevigen, Gaithersburg, MD, USA) as previously described [60]. PBMC DNA was isolated for polymorphism analysis of *PARP1* V762A, *XRCC1* R194W and *XRCC1* Q399R using a commercial DNA purification kit (Qiagen, Germantown, MD), as reported [36].

Paired tumor biopsies were performed in an expansion cohort at MTD pre-and post-cycle 1 for proteomics and apoptosis endpoints (Figure 1). Percutaneous biopsies were obtained by interventional radiologists under CT or ultrasound guidance with independent procedure consent. Samples were processed in real time in optimal cutting temperature compound and stored at −80° C, then cut and stained immediately prior to use as previously described [61]. Optimal tissue quality was defined as paired sequential biopsies with solid tissue areas containing at least 50% tumor cells and less than 25% necrosis [61]. Tissue area was measured as reported [62] and RPPA was performed in 2014 by the MD Anderson RPPA Core facility using their 274-antibody protocol including key proteins for DNA repair [39]. A modified DNA repair score, consisting of expression levels of 11 of 17 DNA repair proteins previously reported to predict PARPi sensitivity in small cell lung cancer in a study by Cardnell et al, was applied (Supplementary Table 1) [40]. Apoptotic cells were counted using the DeadEnd colorimetric TUNEL kit (Promega, Madison, WI, USA) [36]. The apoptotic index was defined as the percentage of TUNEL-positive single cells in five high-power fields.

### Statistical analyses

An expansion group was added at the MTD for exploratory biomarker analyses. For any given endpoint comparison, 10 paired biopsies would provide 80% power to detect a difference between pre- and on-treatment values of one standard deviation of the difference (α_2_ = 0.05). Normalized values for each protein in the RPPA were analyzed for linear correlation to PFS using JMP 9.0 (SAS Institute, Cary, NC, USA). The Hochberg method was used with an alpha of 0.05 to control the FDR [63]. Baseline PAR levels were correlated with response and PFS using the Fisher exact test. PARP1 and XRCC1 polymorphisms were correlated with response and PFS using the Fisher’s exact test and log-rank test, respectively. Pre- and post-cycle 1 treatment TUNEL percent positivity was compared using a two-tailed paired *t*-test (Prism 6, La Jolla, CA, USA), and TUNEL results were correlated with response by Pearson correlation coefficient (JMP 9.0). A p-value of less than 0.05 was considered statically significant, and all tests were two-sided.

## SUPPLEMENTARY MATERIALS



## Abbreviations

MTD: maximum tolerated dose
*BRCA*wt: *BRCA* wild-type
HGSOC: high grade serous ovarian cancer
HR: homologous recombination
HRR: homologous recombination repair
g*BRCA*m: germline BRCA mutation
PARPi: poly (ADP-ribose) polymerase inhibitors
ORR: objective response rate
PFS: progression free survival
DL: dose level
DLT: dose-limiting toxicity
ANC: absolute neutrophil count
PR: partial response
SD: stable disease
PD: progressive disease
CBR: clinical benefit rate
PBMCs: peripheral blood mononuclear cells
TNBC: triple negative breast cancer
HRD: homologous recombination deficiency
