# Supplementary material for: Phase I/Ib study of olaparib and carboplatin in heavily pretreated recurrent high-grade serous ovarian cancer at low genetic risk
Source: Oncotarget. 2019 Apr 23;10(30):2855–68. doi: 10.18632/oncotarget.26869 (PMC6499601; doi:10.18632/oncotarget.26869)
Supplement: Supplementary file 1 [file oncotarget-10-2855-s001.pdf]

Phase I/Ib study of olaparib and carboplatin in heavily pretreated recurrent high-grade serous ovarian cancer at low genetic risk

SUPPLEMENTARY MATERIALS

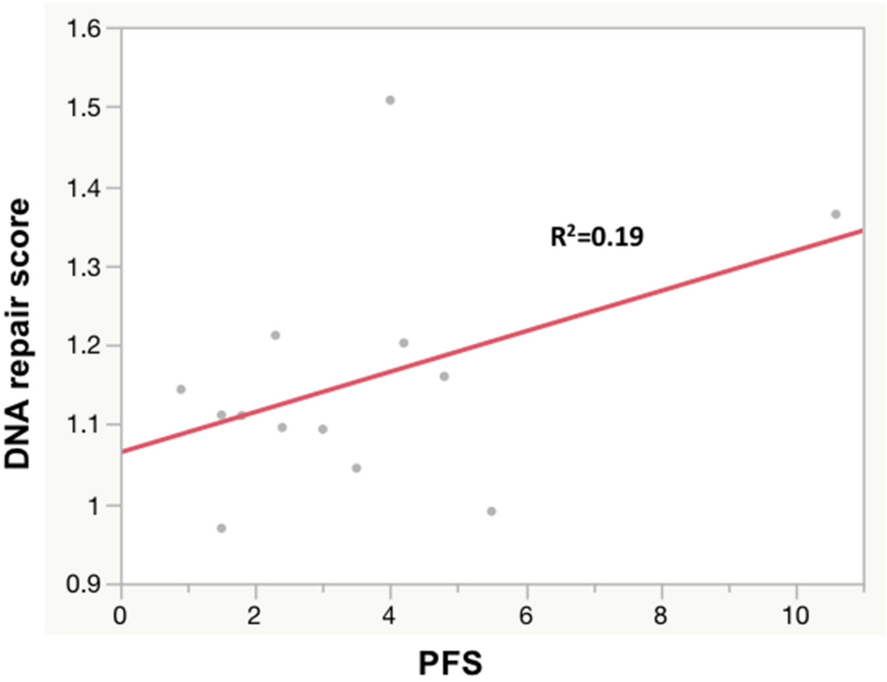

Supplementary Figure 1: DNA repair score by progression free survival. There was no correlation between DNA repair score and PFS. Abbreviations: PFS = progression free survival.

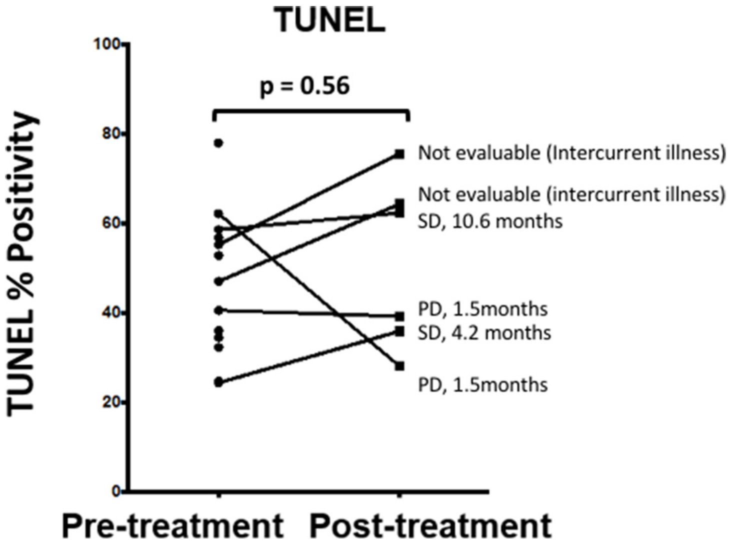

Supplementary Figure 2: Tissue apoptotic index. There were no significant differences in tissue apoptotic index before and after cycle 1 treatment with combination therapy of olaparib capsules and carboplatin.

Supplementary Table 1: 17 Proteins in the original "DNA Repair Score"

**pChk1**	**ATM**
FANCD2	**RAD50**
MRE11	NBS1
**pChk2**	**MSH2**
**Chk2**	**PARP1**
**pATM**	**RAD51**
**XRCC1**	BRCA1
**53BP1**	ATRIP
DNA.PKcs	

Bolded proteins were included in our modified DNA repair score.
